# Perceptions of Yoga Therapy Embedded in Two Inpatient Rehabilitation Hospitals: Agency Perspectives

**DOI:** 10.1155/2015/125969

**Published:** 2015-09-30

**Authors:** Marieke Van Puymbroeck, Kristine K. Miller, Lori A. Dickes, Arlene A. Schmid

**Affiliations:** ^1^Department of Parks, Recreation, and Tourism Management, School of Health Research, Clemson University, Clemson, SC 29634-0735, USA; ^2^Department of Physical Therapy, School of Health and Rehabilitation Sciences, Indiana University, Indianapolis, IN 46202-5119, USA; ^3^Clemson University, Strom Thurmond Institute, Kappa Drive, Clemson, SC 29634, USA; ^4^Department of Occupational Therapy, College of Health and Human Sciences, Colorado State University, Fort Collins, CO 80523-1573, USA

## Abstract

Inpatient medical rehabilitation has maintained a typical medical-model focus and structure for many years. However, as integrative therapies, such as yoga therapy, emerge as treatments which can enhance the physical and mental health of its participants, it is important to determine if they can be easily implemented into the traditional rehabilitation structure and milieu. Therefore, the purpose of this study was to examine the perceptions of key agency personnel on the feasibility and utility of yoga therapy implemented in inpatient rehabilitation. This study reports the results of focus groups and an individual interview with key stakeholders (administrators and rehabilitation therapists) from two rehabilitation hospitals following the implementation of yoga therapy. Results focused on several key themes: feasibility from the therapist and administrator perspectives, challenges to implementation, and utility and benefit. Overall, the implementation and integration of yoga therapy were positive; however, some programmatic and policy and organizational considerations remain. Implications for practice and future research are provided.

## 1. Introduction

The International Association of Yoga Therapists (IAYT) defines yoga therapy as “…the process of empowering individuals to progress toward improved health and well-being through the application of the philosophy and practice of Yoga” [1] (p. 3). Yoga therapy is available in the community and has been shown to improve physical [2–8] and mental [3, 4, 7–11] functioning with individuals with chronic disease in the community. However, to our knowledge, yoga therapy has not been integrated into inpatient Western medical rehabilitation (known henceforth as rehabilitation). Inpatient rehabilitation is for individuals who require additional time to recover and repair from an acute trauma to the body following hospitalization, ranging from elective joint replacements to catastrophic brain or spinal cord injury. Rehabilitation is often provided by several therapeutic disciplines, including physical therapy, occupational therapy, recreational therapy, speech language pathology, and rehabilitation nurses.

Theoretically, the structure of yoga therapy could be easily integrated into rehabilitation. As Elgelid [12] identified, yoga therapy can help with Phase 1 of rehabilitation (acute injury) through the use of pranayama (breathing techniques), pratyahara (withdrawal of the senses), and dhyana (meditation) by reducing pain and increasing focus on body awareness. Yoga therapy can also assist with Phase 2 of rehabilitation (repair) by focusing on yamas and niyamas (rules for living), dharana (concentration), and asanas (physical postures). In Phase 2, these components of yoga therapy will help the body and tissues repair. In the final phase (3; Remodeling), the focus should be on asanas (physical postures) in order to restore function to the highest possible level [12].

Taylor [13] stated, “the interface between yoga therapy and rehabilitation is fertile ground for substantive health reform, each informing and shaping the other” (p. 94). While this is a lofty goal, changing or enhancing the existing structure of rehabilitation in the United States is a challenge, due to numerous and extensive payment and insurance-related regulations. Therefore, the purpose of this study was to examine the perceptions of key agency personnel on the feasibility and utility of yoga therapy implemented in inpatient rehabilitation.

## 2. Materials and Methods

Yoga therapy was embedded into two inpatient rehabilitation units in the Midwest United States for people recovering from acute injuries, including spinal cord injury, traumatic brain injury, and stroke to determine if it was feasible and useful to incorporate as a part of care, both from a patient perspective and agency perspective. This study reports the findings from the agency perspective; the patient perspectives are reported elsewhere [11].

Patients at the rehabilitation hospitals were eligible for the study if they were able to speak and understand English, score at least 4/6 on the 6-item Mini Mental State Exam, and were at least 18 years old. Patients were not eligible for this study if they were dependent on oxygen, were participating in a research study, or had a diagnosis of a serious cardiac condition or drug/alcohol abuse.

Eligible participants were able to choose group or individual yoga sessions (or both) and received a guided relaxation audio recording. All yoga sessions were offered twice weekly and added to the participant's rehabilitation daily schedule. The yoga sessions were led by a certified yoga therapist, who modified the postures as necessary for each participant. Group yoga classes were 45-minute sessions, while individual yoga classes lasted for 30 minutes. Yoga asanas, or postures, occurred in a chair, wheelchair, or bed, as needed. For individuals who were unable to perform physical asanas, the practice focused on pranayama or breathwork. Research assistants were available to assist with modifications of physical postures as needed. Participants used the guided relaxation as they desired.

Following the three months during which yoga was offered at the inpatient rehabilitation hospitals, qualitative interviews were employed with key stakeholders at the rehabilitation hospitals to determine the feasibility and utility of embedding yoga therapy into ongoing therapy. Thus, three focus groups were conducted with two rehabilitation hospital administrators (research coordinator and nurse director), two therapy directors, and five rehabilitation therapists (two physical therapy assistants, one physical therapist, one occupational therapy assistant, and one occupational therapy assistant student). The individual interview was conducted with a physical therapist who was unable to attend the focus groups due to scheduling conflicts. These individuals were chosen for the focus groups and interview based upon their involvement in scheduling patients for yoga therapy, their awareness or management of yoga therapy implementation in their clinics, or the amount of patient care they provided daily to the patients. This study was approved by the local institutional review boards.

The questions for the focus groups and interview were open-ended but structured to elicit information related to the research question. For example, questions included the following: “Describe how the implementation of yoga therapy was perceived by staff?” “What problems were experienced by staff regarding the implementation of yoga therapy?” “What were the benefits to the patients of yoga therapy?” “What was your perception of the usefulness of yoga therapy to your patients?” “What was the downside for implementing yoga therapy on the unit?” “What are your thoughts on scheduling yoga therapy for patients in an inpatient rehabilitation setting?” Probes were used with each question to elicit more information.

### 2.1. Data Management and Analysis

All focus groups and the interview were audio-recorded and transcribed verbatim. Content analysis was used to analyze the data. Content analysis is a fluid method of analyzing text data [14] and focuses on the content or meaning of the text [14, 15]. In qualitative content analysis, large quantities of data are classified into categories or themes that have similar meanings [16]. More specifically, content analysis is “a research method for the subjective interpretation of the content of text data through the systematic classification process of coding and identifying themes or patterns” [14] (p. 1278).

This study utilized conventional content analysis; that is, themes were determined from the content of the text [14]. After a thorough reading of all of the data, words in the text that provided a solid example of a key concept were highlighted. The data were read repeatedly and the researcher kept notes of impressions of the data and began to develop initial analyses based on these notes. During this process, the notes, impressions, and analyses were coded into meaningful clusters of related content [14]. Next, each cluster was examined for content, and a definition of the theme for the cluster was developed [14]. Direct quotes, considered to be exemplars for each cluster, were identified and are included in the results for clarity. Two authors reviewed the themes independently (MVP and AAS) and then compared their categorizations. There was 95% agreement initially, and following the discussion of individual theme categorization, the authors were able to agree on the remaining 5% discrepancy.

## 3. Results 

The themes that emerged from the data were feasibility (with subthemes of therapist and administrator perceptions; challenges of implementation) and utility and benefits. Each theme is explored below.

### 3.1. Feasibility

Rehabilitation units in the United States require that patients participate in at least three hours of physical, occupational, and speech therapy per day. In addition, other therapies, such as recreational therapy and nursing, also provide scheduled care for the patient. Scheduling a patient for these therapies is often already a challenge, and adding in a new type of therapy may present additional difficulties. The therapists in this study identified that the stress of the three hours of required therapy is taxing on patients, and adding another hour for yoga therapy resulted in more exhaustion for the patients. They suggested that if the sessions were offered first thing in the morning, it may help the patients better prepare for the day.

Both rehabilitation hospitals had a morning “huddle” at which each patient and their daily schedules are discussed. The hospitals handled scheduling differently, but each scheduled the patients for yoga therapy in the same matter as the patients were scheduled for the other therapies. This appeared to be a key in ease of scheduling the patients for yoga therapy, and the therapists and administrators both spoke highly of this process. As one administrator stated, “And, we're pleased you've really integrated kind of seamlessly, kind of wormed your way in there when patients were not in therapy or whatever… yours is really the first, what do I want to say, actual physical intervention that was worked in around their schedules.”

Another feasibility issue occurred in terms of space to provide the yoga therapy intervention. Typically, yoga therapy is conducted in a quiet space that has little environmental interruptions. Typical rehabilitation treatment areas are noisy and busy places with numerous people on the move, and space is hard to find. Staff from both facilities described having trouble finding an appropriate location for the yoga therapy intervention. At one facility, the yoga therapy was done in or near the lobby, which was the quietest location available (albeit not very quiet). At the other facility, the multipurpose room, which was the domain of the recreational therapist, was used. There was some concern about territorial issues with this room, but the yoga therapy interventions did not conflict in timing with recreational therapy, and thus there were no scheduling issues.

### 3.2. Therapist Perceptions

The therapists identified that they were “pleasantly surprised” about the implementation of yoga therapy into the rehabilitation milieu. They described feeling that the yoga was holistic and appreciated that it addressed the physical, social, and mental needs of the patients. As one therapist stated,

I think with the level that [the yoga therapist] brought to these patients; I thought it was great. Um, I really… it's easy to be as a physical therapist (PT) or even as an occupational therapist (OT) and to go in and just hone in on your area, but in the rehab realm you really have to take all sides of consideration into their health. And, so to not just say “well I'm a PT so I just need to work on walking and this and your strength” and then to be able to tailor it and bring other aspects into the picture that are important to them for their safe discharge home. I really thought it was great to incorporate the level she did.

Another therapist spoke of the benefits of yoga as a holistic approach:

I really liked the, like I had mentioned, the holistic approach that that had brought on. So, bringing that aspect where, um, we don't always have time to, um, utilize that in our intervention or even just to be able to, uh, teach them some of those basic breathing and relaxation techniques. Um, I just thought that was a great adjunct to that program as a whole.

The therapists also reported that the breathing techniques relaxed the patients with a lot of anxiety, which helped to center them and resulted in better outcomes. Therapists also suggested that getting patients out of their room for yoga therapy was beneficial, particularly because of the ability to do yoga therapy with a group. One therapist stated,

…I mean the individual [session] is important but I think I could see it more as an adjunct to doing groups. You know, because we don't do those things anymore. We used to have a lot of groups. It was a good source of camaraderie and getting to know other people.

Furthermore, therapists suggested that having therapists explain it to the patients was important. As one therapist stated,

One thing is I think the perception of yoga also affects how you promote it to your patients. Like because I found sometimes with my patients have always wanted to do yoga, but they are in this state, but they don't see themselves doing stretching and all those things. So, I would explain to them there are different options; you know you could focus on breathing, it can help you. Sometimes I find like people might not be into real yoga, stretching and all those things; they were just interested in the breathing part, some of them were interested in the actual yoga part. So, kind of explaining the difference was helpful, and some of them just didn't want to do it. Most of them, most of the reviews I got, always said I've always wanted to try it. I would like to try it at least once to see how it would work for me.

### 3.3. Administrator Perceptions

The administrators had a different view of the implementation of yoga as they interacted with nursing and therapists. The administrators at one site identified that nursing staff felt left out of the planning and information sessions about the yoga therapy intervention. Specifically, these information sessions should focus on the benefit of yoga therapy for the patient, because as one administrator stated:

So, I do think they [the nurses] are open, but I think they would be open to anything in my opinion especially if it's something that would benefit the patients. Um, you know, obviously with pain management that's a huge focus for us in spinal cord. Um, stress, dealing, coping, those types of issues. So, yeah, I do think the receptivity is there, but again, I think they are lacking some education around it on the basics. Like you said, if you mention research to a lot of the staff, they… they blank out on you.

The administrators also recommended that future yoga therapy interventions involve the nurses and that researchers have sessions about the interventions specifically for the nurses in order to enhance overall communication between the research and the treatment team. The administrators also suggested that inviting the nurses to attend or observe some yoga therapy sessions would help to make nursing feel more a part of the process. At the other site, nurses asked if they could sit in on the yoga therapy sessions. An administrator stated that she, “… did mention to them [the nurses] that they were more than welcome to observe the session. And, certainly [the yoga therapist] was very approachable; that they could ask questions.” At this site, there did not seem to be the same perception of a lack of communication; thus, it is possible that incorporating nurses into the yoga therapy makes a difference in the perceptions of feasibility for yoga therapy.

The administrators spoke of the yoga therapy intervention aligning with the mission at their respective hospitals, which both addressed healing the mind, body, and spirit of the individual.

### 3.4. Utility and Perceived Benefit

In current times and due to reducing resources in health care, territorialism is sometimes an issue on rehabilitation units [17]. The therapists and administrators were questioned about their perceptions of the utility and benefit of embedding yoga therapy into the rehabilitation unit. As one administrator stated, “the patients I have run into have just raved about it. They loved it.” The patients told the therapists and administrators about benefits related to relaxation and enjoyment. The patients reported that the breathing aspect of yoga provided specific relaxation, particularly for those with high-level spinal cord injury. As one therapist stated,

Most of my patients; I referred for relaxation and those things and they kind of seemed to benefit from that. Especially breathing techniques; they were all very pleased with those things. Just learning you know, about how to focus on breathing, how to calm themselves down and maintain stress and anxiety. So, those things are the things that seemed to help.

A therapist spoke of the yoga therapy being tremendously helpful for a patient with anxiety about mobility. She stated

That was something he just found of the most benefit because he said he can do his posture and breathing and relaxation and then it helped him walk through the steps of what he needed to do to transfer over to the other tasks.

Thus, the perception from therapists was that yoga provided skills that were able to assist in improving other aspects of rehabilitation.

A therapist also spoke of the qualities of the yoga therapist, which also put patients at ease and may have contributed to the positive effects. She stated,

I do know the few times that I observed, um, [yoga therapist] working with these patients, that um, she obviously has a wealth of knowledge in that field and was, um, certainly, um, a great person to lead these groups or these, you know, this intervention with these patients. So, I thought that was great. She was very quick to build a rapport with patients, and was very comfortable coming in and doing what she needed to. That was wonderful to see. So, that the patients were receptive to her and very appreciative of her information and just her friendliness as a whole—they really enjoyed that. So, I know that was some of the things they had mentioned as well.

Overall, the therapists and administrators identified that adding yoga therapy to inpatient rehabilitation was a positive experience and was not intrusive to the rehabilitation milieu. Both groups identified that embedding research studies, like this one, into the rehabilitation hospital is important and had relatively little negative impact on their unit.

## 4. Discussion

Yoga therapy was successfully implemented into two inpatient rehabilitation hospitals and studied for its feasibility and implementation potential. This study illustrates some of the policy and organizational challenges of implementing new therapies and treatments across the broader U.S. health care system. In this case, the results illustrate a classic principal agent dilemma. At its most basic this relationship assumes that the agent, that is, “therapists” or “nurses” (or anyone that is employed by the rehabilitation hospital), will operate on behalf of the principal, or in this case “the administrators.” The relationships assume that when new agents are asked to implement new techniques they are trusted to implement and follow through on these efforts. However, principals and agents are often motivated by different primary objectives and, as such, may have conflicting ideas of the value and feasibility of new therapies or treatments [18].

In terms of feasibility, one key issue was that the timing of the therapy is important. Some stakeholders felt that adding an additional hour of therapy to patient's daily load of three hours made it difficult for maximum participation. Determining if the additional hour of therapy was beneficial to the patient's physical and mental health would be important for future studies. The therapists and administrators suggested that by having yoga therapy as one of the daily scheduled therapies, scheduling was fairly smooth overall. Appropriate space to implement yoga therapy is also a consideration, as rehabilitation units tend to be loud, busy places.

This type of health care intervention underscores the importance of organizational barriers, like physical space and time, in implementing new treatments. This concern of stakeholders further highlights the principal agent challenge [18]. The agents, nurses and other therapists, may believe in the treatment value of a therapy and even lobby for its implantations. However, if the principal is not willing to allocate resources to overcome the specific barriers, the treatment may not be adopted. With regard to additional physical space or changing the allocation or professional's time, a principal may be reluctant or not have the budget allocation to make this change no matter how beneficial agents believe a therapy is.

The therapists perceived that yoga therapy was a positive adjunct to the traditional therapies offered, primarily because it is holistic and addressed the physical, mental, and social needs of the patients. In terms of increasing attendance at yoga therapy, the therapists noted that it was important for the other therapists to support and encourage patients to attempt yoga therapy. This is supported by recommendations from Wurz et al. [19] who identified that physician and therapist referrals to yoga therapy are important and may be impeded by lack of knowledge of the benefits. This could be a key for future implementation of yoga therapy in these settings. The therapists also discussed the benefits of yoga therapy being conducted in groups, where many of the other therapies are no longer delivered in that fashion. Numerous studies have identified that the social support gained from group yoga therapy is beneficial in helping patients feel connected to others and not isolated because of their disability or health condition [8, 19–21].

Administrators noted some communication problems surrounding the implementation of the study, the daily yoga therapy, and the interaction with the nurses. Long and colleagues [22] identified that nurses often felt undervalued and did not perceive reciprocity with rehabilitation team members. This may have led to the nurses at one site not feeling aware of the benefits of yoga therapy and the need to implement yoga therapy in this setting and not feeling involved or aware of yoga therapy. At the other site, nurses asked to be involved, and there were no communication issues. These results illustrate a key to any policy transition, that is, transparent communication and broad stakeholder buy-in and communication.

However, in this case not just nurses need this information; it is important that all team members be aware of the benefits of yoga therapy. As Wurz et al. [19] noted, it is essential to determine the specific benefits of the yoga therapy on the intended population and to share that with the health care professionals who work in the program. Nurses, particularly, are the primary liaisons for care for each patient in terms of assessment, coordination, therapy scheduling or facilitation, and the provision of care and support [22], and it would have likely been better to make sure the nurses were actively involved. Wurz et al. [19] also support involving health care professionals, such as nurses, through education in rounds (or huddles, as was the case at the two rehabilitation hospitals in this study) and by having yoga therapists around to talk directly with the staff. Pellatt [17] identified that when the care staff communicate effectively, it is likely that better and more coordinated care is provided to the patients. This analysis further confirms the importance of clear communication and support from rehabilitation nurses, and other staff, if implementation of yoga therapy is to be successful.

The therapists and administrators both perceived that this study was useful and had benefits to the patients. The therapists also identified that the breathing exercises associated with yoga were particularly beneficial, as it was something patients could control, even if they had control over little other movement in their body. This extended into perceptions that yoga helped to reduce the anxiety of their patients, which allowed patients to handle the inpatient experience a little better. These perceptions are supported by numerous studies on the power of yogic breathing techniques on anxiety and control in individuals with disease or disability [10, 23–28].

As with any study, there are limitations to this study. This study implemented yoga therapy at two Midwestern rehabilitation hospitals, and this is not necessarily representative of rehabilitation at other hospitals in other parts of the United States or in countries where rehabilitation is practiced differently. This study was limited also by scope, as outcomes from the intervention focused on patient and stakeholder perceptions of feasibility and utility, not functional improvement. Preliminary feasibility and utility were established with this study; however, future research should consider embedding yoga therapy in a rehabilitation hospital for a set period of time to determine payment for services and enhanced functional outcomes, comparing these data with a control group. Additional research should also incorporate perceived policy and organizational challenges to understand the needs for implementation success.

## 5. Conclusion

In conclusion, in the stakeholder's opinions, it was feasible to implement yoga therapy in inpatient rehabilitation settings. These stakeholders reported perceiving that yoga was useful to the patients and it provided an added benefit to rehabilitation patients. By addressing the concerns that arose during this process, it appears that yoga therapy could be implemented in inpatient rehabilitation settings and potentially enhance the rehabilitation experience for patients by developing self-management skills and wellness behaviors to support long-term recovery. Additional research with control groups and a broader understanding of organization challenges will provide a stronger foundation for implementing rehabilitation yoga programming more comprehensively.

## Appendix

### Focus Group Questions

1. Describe how the implementation of yoga therapy was perceived by staff.
2. What problems were experienced by staff regarding the implementation of yoga therapy?
3. What were your perceptions of the benefits to the patients of yoga therapy?
4. What was your perception of the usefulness of yoga therapy to your patients?
5. What was the downside of implementing yoga therapy on the unit?
6. What are your thoughts on scheduling yoga therapy for patients in an inpatient rehabilitation setting?
7. Did yoga therapy interfere with the services you provide? Please describe the integration of yoga therapy with the other therapies.
8. In your opinion, how did scheduling patients for yoga therapy work? What could have made it work better?
9. How do you think yoga therapy fits in with the mission of [hospital name]? Describe.
10. How did the administrators perceive the yoga therapy?
11. How did the therapists perceive yoga therapy?
12. What helpful hints or suggestions do you have for us as we think about making inpatient rehabilitation a part of regular programming?
13. What other information would you like to provide us how you perceived the yoga intervention or any thoughts about the yoga in general?
